# The psychological consequences of living with coronary heart disease: Are patients' psychological needs served? A mixed‐method study in Germany

**DOI:** 10.1111/hex.13467

**Published:** 2022-10-21

**Authors:** Samia Peltzer, Ursula Köstler, Hendrik Müller, Nadine Scholten, Frank Schulz‐Nieswandt, Frank Jessen, Christian Albus, The CoRe‐Net Study Group

**Affiliations:** ^1^ Department of Psychosomatics and Psychotherapy, University Hospital Cologne, Faculty of Medicine University of Cologne Cologne Germany; ^2^ Department of Social Policy and Methods of Qualitative Social Research, Faculty of Management, Economics and Social Sciences University of Cologne Cologne Germany; ^3^ Department of Psychiatry and Psychotherapy, University Hospital Cologne, Faculty of Medicine University of Cologne Cologne Germany; ^4^ Institute for Medical Sociology, Health Services Research, and Rehabilitation Science (IMVR), Faculty of Medicine and University Hospital Cologne, Faculty of Human Sciences University of Cologne Cologne Germany; ^5^ German Center for Neurodegenerative Diseases (DZNE) Bonn Germany

**Keywords:** coronary heart disease, healthcare system, mental health issues, mixed‐method approach, value‐base healthcare

## Abstract

**Introduction:**

This mixed‐method study explores psychological needs, access and barriers in coronary heart disease (CHD) patients with and without mental health issues (MHI) within the German healthcare system.

**Methods:**

This study was conducted in three different healthcare settings: two hospitals, two rehabilitation clinics and three cardiology practices in Cologne, Germany. Patients were screened for angiographically documented CHD and other inclusion criteria. In total, 364 CHD patients took part in this study. It consisted of two parts: In the first part, participants filled in a newly developed questionnaire about their psychological needs, access and barriers within the healthcare system and their contact with their doctor in these matters. Then, patients were screened for MHIs with the help of the Hospital Anxiety and Depression Scale (HADS). When a score above seven was scored on the HADS, patients were additionally screened for specific MHIs using the Structured Clinical Interview for DSM‐IV Axis I Disorders. In the second part, 20 participants were subsequently interviewed in a semi‐structured interview to generate more in‐depth findings.

**Results:**

The interviews show that CHD patients with and without MHI experienced a cardiac event as life‐changing and had an urgent need to talk about CHD with their doctor, mostly the general practitioner (GP). When the GP spoke to the patient shortly after the cardiac event, patients experienced relief and were better able to cope with their illness. Only 9.1% reported being aided in their search for psychotherapeutic treatment or drug treatment (4.1%).

**Conclusion:**

The needs of CHD patients with and without MHI were not adequately satisfied within our sample. Psychological measures are necessary for sufficient improvement, such as training of doctors in doctor–patient communication (e.g., better support in coping with MHI/CHD), improvements in the procedure (more time for conversations during doctor contacts), and improvement of structural requirements (referring patients faster to psychotherapists).

**Patient or Public Contribution:**

We received input from patients during pretests and used the feedback to tailor our questionnaire and the interview guidelines. Afterwards, we disseminated the main results for the patient and public involvement (e.g., public lectures, leaflets for self‐help groups, etc.).

## 1 INTRODUCTION

### 1.1 Coronary heart disease

Coronary heart disease (CHD) is the leading cause of mortality, accounting for approximately one‐third of all deaths in individuals over the age of 35 years, with a death rate of 102.6 per 100,000.^1^ In Germany, the lifetime prevalence of CHD for the age group 40−79 years is 9.3% (8.4−10.3).^2^ Short‐term mortality has been decreased due to advances in acute CHD treatments. However, at a population level, CHD morbidity has increased in recent years. After an interim decline in smoking, hypercholesterolaemia and hypertension, these currently appear to be on the rise again.^1^, ^3^, ^4^

### 1.2 Mental health issues and their relationships with CHD

It has been found that mental health issues (MHIs) are highly prevalent among patients with CHD. MHI is defined as a recognizable set of clinical symptoms and/or behavioural problems that correlate with individual distress and impairment of functioning at the individual level.^5^ After a myocardial infarction, almost 30% of these patients suffer from depressive symptoms and 20% even fulfil ICD‐10 criteria for a depressive episode.^6^ Depression is associated with higher CHD‐related morbidity and mortality (relative risk = 1.6–2.4): After a cardiac event such as a myocardial infarction, CHD patients with comorbid depression have twofold greater mortality during the next 2 years.^7^ Furthermore, mortality risk can increase sixfold when the patient suffers from severe depressive symptoms.^8^

### 1.3 MHIs as barrier to medical adherence

MHI also acts as a strong barrier to treatment adherence and exacerbates a required lifestyle change that is necessary to reduce CHD risk factors—regardless of whether MHI were already pre‐existing or occurred as a consequence of CHD.^4^ For example, patients with MHI are less likely to exhibit healthy lifestyle behaviour (e.g., quit smoking) and have low medication adherence.^9^ This emphasizes the drive for more patient involvement in terms of disease management and medical consultations.^10^ At the same time, this group of patients finds it difficult to understand the course of the consultation and to present their own concerns adequately, resulting in a lack of communication of possible physical and/or MHIs to their doctor.^11^ Hence, this specific population of CHD patients with MHI may be very vulnerable due to difficulties in disease management, low communication skills in a medical setting and problems communicating possible issues and questions about personal topics. This suggests that the quality of communication between patients and their doctors is important to secure the best course of treatment.^12^

Consequently, adequate screening and treatment for MHI in CHD patients during the medical consultation from the doctor's perspective is urgently needed. National and international guidelines have recommended routine screening and treatment for MHI on primary and secondary CHD prevention.^4^, ^13^ However, studies have been able to show that this screening is not regularly followed due to lack of time and low occurrence of verbal interventions.^14^, ^15^

### 1.4 Current study

In the current study, we explore the following research questions: (1) What are the MHI‐related needs of CHD patients with MHI after a cardiac event?, (2) how well do doctors meet their patients' needs? and (3) how do patients experience access and barriers to the healthcare system? It is hypothesized that patients with MHI have a desire for doctors to approach and support them with their MHI. Secondly, it is expected that doctors rarely meet their patients' needs. Lastly, it is hypothesized that, due to Hypotheses 1 and 2, patients receive a lack of access and distinct barriers to the healthcare system, especially related to their MHI. The aim of the study is to improve the quality of care and initiate changes in the structure of healthcare towards more patient‐centred care in the long term.

## 2 MATERIALS AND METHODS

This study (MenDis‐CHD) is one of three projects carried out within the framework of the Federal Ministry of Education and Research (BMBF)‐funded Cologne Research and Practice Network (CoRe‐Net). MenDis‐CHD was approved by the Ethics Commission of Cologne University's Faculty of Medicine (Committee Reference Number: 17–220) on 26 September 2017.

### 2.1 Design

MenDis‐CHD is a cross‐sectional study with a mixed‐method approach using an explanatory sequential design,^16^ beginning with quantitative data collection (one questionnaire containing: questions about sociodemographic data, patients' needs and access and barriers to the healthcare system; psychological assessments [Hospital Anxiety and Depression Scale {HADS}, SCID‐I]), followed by a qualitative data acquisition (semi‐structured in‐depth interviews) to enrich the quantitative data. Specific details of the methods used in this study can be found in an earlier paper.^17^ The questionnaire was fully developed in German. Individual translated items can be provided upon request.

### 2.2 Patient contribution

We received input from CHD patients during the pretests of the questionnaire and the qualitative interviews, as well as from members of CHD self‐help groups. The patient perspective has been continuously taken into account in the project and the interests of the patients have also been considered with regard to the transfer of results (e.g., through public lectures, summaries in plain language and design of a leaflet to share with peers and distribute to patient groups). Patients did not receive any compensation for contributing to this study.

### 2.3 Participants

As inclusion criteria, patients had to have an angiographically documented CHD with stable angina pectoris, acute coronary syndromes, percutaneous coronary intervention (PCI) or bypass surgery, be older than 18 years, have sufficient German language skills and be able to give informed consent. Severe physical (e.g., cancer) or unstable mental problems (e.g., acute suicidal ideation, delirium and moderate to severe dementia) were the exclusion criteria.

**Figure 1:**
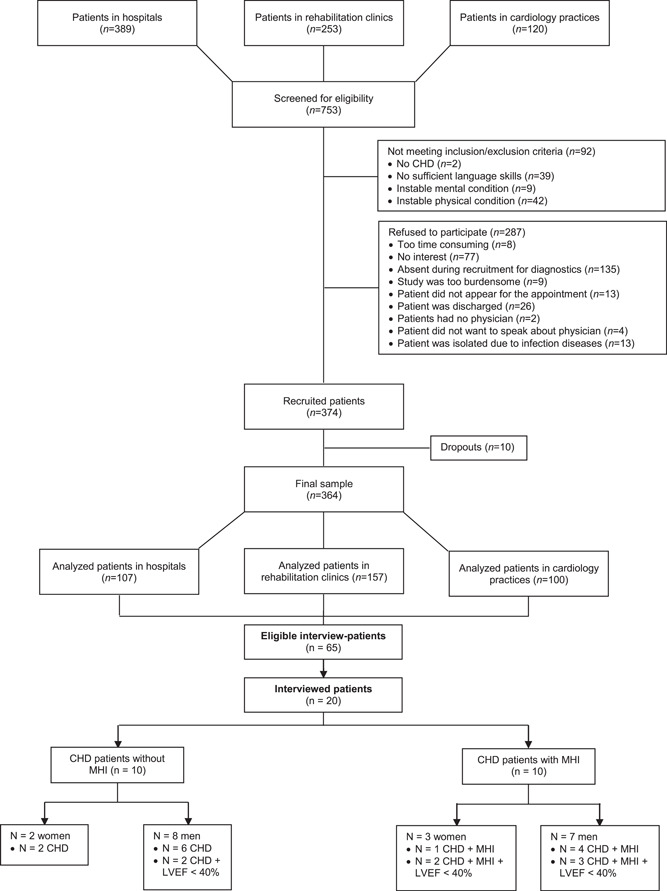
Flowchart of the recruitment procedure. CHD, coronary heart disease; LVEF, left ventricular ejection fraction; MHI, mental health issue

Overall, 753 patients were screened for eligibility, of whom 374 were finally enroled in the study. Ten patients dropped out due to withdrawal of informed consent or incomplete questionnaires with missing answers. Therefore, 364 patients were included in the final data analysis. We recruited 107 patients in hospitals, 157 patients in rehabilitation clinics and 100 patients in cardiology practices. Figure 1 features a flowchart presenting the recruitment process.

All 364 included patients filled in the questionnaire. We used maximum variation as the sampling strategy to increase diversity by age, gender, marital status, employed/retired status, severity of CHD, left ventricular ejection fraction and kind and severity of diagnosis of MHI.^18^ By explicitly aiming for recruiting a diverse sample, one ensures the generalizability of one's findings across a broader part of the general population. Furthermore, we asked every eligible and interested patient during the recruitment phase to also participate in our in‐depth interviews. In total, 20 patients were selected to participate in the qualitative interviews. The diversity of participants was actively monitored, but due to a significant number of patients who did not want to participate in the interviews, participant diversity was not completely balanced, especially gender and ethnicity. In this study, the sample consisted of approximately 70% males. According to the current literature, males are significantly more likely to develop CHD,^19^ with a lifetime prevalence of 9.9% (9.0–10.8) than women, with 6.7% (6.0–7.4).^20^ Thus, this study seems to represent an appropriate sample population. For further details about the distribution of samplings for interview groups, see Figure 1.

### 2.4 Research tools

#### 2.4.1 Quantitative

Sociodemographic data were assessed via a newly developed questionnaire. Disease severity of CHD patients was assessed by screening for cardiac events in previous medical history (e.g., myocardial infarction), cardiac surgeries (e.g., bypass surgery), congestive heart failure, somatic comorbidity, pre‐existing presence of MHI, left ventricular ejection fraction and New York Heart Association stage (classification scheme of heart diseases according to their degree of severity). All of these characteristics were drawn from patients' medical records; they were not part of the questionnaire. Clinical data were measured with the use of the HADS and SCID‐I.

An aforementioned self‐made questionnaire was made of five subsections of which two are used in this paper, namely ‘Patients' needs’ and ‘Access and barriers to healthcare system’. The questionnaire was developed and used in the German language only; all items and section headers provided in this article are translated.

Both sections, ‘Patients' needs’ and ‘Access and barriers to the healthcare system,’ were assessed by a 20‐item self‐report questionnaire. Patients without MHI could skip several items that contained questions about an existing MHI. The section ‘Patient's needs’ contained six questions, such as ‘Do you wish to be approached by the doctor on possible mental health issues?’ with binary answer possibilities (Yes/No) or multiple‐choice questions such as ‘Who do you think should give a recommendation for treatment in the case of mental health issues?’. The section ‘Access and barriers to healthcare system’ contained 14 questions, also designed with ‘yes or no’ or multiple‐choice questions such as ‘How often have you sufficiently received information on the content and accessibility of psychological care?’. The questionnaire was developed within this study with the help of four CHD patients with and without MHI and has yet to be validated. More information on the content of the questionnaire and how it was created can be found in our first publication on MHI.^21^

The HADS^22^, ^23^ was used as a general screening tool to assess symptoms of depression and anxiety and was filled in by the patients. In line with the advice of several studies,^24^ a score of 8 and above was considered a ‘positive’ test result for anxiety or depression. In this case, the Structured Clinical Interview for DSM‐IV Axis I Disorders (SCID‐I)^25^ was conducted as a semi‐structured interview to assess mental symptoms and disorders as defined in the ICD‐10.^6^

#### 2.4.2 Qualitative

The qualitative module was designed to build on the quantitative part in a second phase to enrich the quantitative findings and further investigate the research questions of the study.^16^ The qualitative study design was conducted according to the method of the problem‐centred interview.^26^ This is a semi‐structured in‐depth interview approach that allows the respondent to speak as freely as possible but is centred on (a) predefined problem(s) to which the interviewer repeatedly refers back during the conversation. Based on the research questions of this study, three main categories were developed: (1) *Category* ‘Perception of the cardiac event and approaches of doctors to talk about MHI’: Were CHD patients approached by the doctor on MHI and after a cardiac event? How did patients experience this approach? What were their expectations? What were positive and negative aspects of the contact with the doctor? Example questions were ‘Have you been approached by the doctor after a cardiac event and if so, how did you experience it? How long did it take until you were asked? Which aspects have been discussed during your meeting? What were your expectations beforehand and what are your expectations now?’; (2) *Category* ‘Patient's needs’: Do patients want to talk about MHI? Who do patients want to talk to about MHI? If a doctor is the first choice, what type of doctor? Example questions: ‘Who asked you about MHI? Who would you have liked to be addressed by? What did you need (both physically and mentally) after you received your diagnosis?’; (3) *Category ‘*Access and barriers’: How exactly do patients experience access and barriers to the health care system? Example questions: ‘What information and offers of help were accessible? How well could then be implemented? What information or offers did you receive, which did you not receive albeit your wish to receive them?’

### 2.5 Procedures

#### 2.5.1 Quantitative

The questionnaire was pre‐tested on two persons with CHD, one with MHI and one without MHI, in July 2017. Participants were recruited between 15 January 2018 and 29 March 2019 in two hospital cardiac departments, three cardiology practices and two rehabilitation clinics in Cologne, Germany, to achieve an appropriate realistic sample of routine healthcare settings. After screening for eligibility through the researchers, all patients' demographic data were documented in our screening log. Those who fulfilled our inclusion criteria and provided written informed consent received the quantitative questionnaires. A second appointment was arranged to perform the SCID‐I if the HADS screening was ‘positive’. All researchers of this study were trained in applying the SCID‐I and experienced in conducting it. In addition, patients were asked to participate in qualitative interviews.

#### 2.5.2 Qualitative

The interview guideline was pretested by two CHD patients: one with MHI and the other without. Pretests were conducted as face‐to‐face interviews in September 2018. Out of 65 participants who agreed to take part in a qualitative interview, 20 were randomly selected. The actual 20 interviews were carried out as face‐to‐face interviews between October 2018 and March 2019, either during a home visit or at the setting where the respective patient was recruited. Informed consent was obtained during the quantitative phase, but the interviewer informed all patients again by reading the information sheet and informed consent to them to ensure that they fully understood each element. The interview took 1 h. All interviews were audio‐recorded and transcribed.

### 2.6 Data analysis

#### 2.6.1 Quantitative

All presented data were analysed using IBM SPSS Statistics 22 software^27^ and controlled for both missing and impossible values. Identified cases with either missing or implausible values were compared to their equivalent paper versions and corrected. Multivariate analyses of variance and *χ* ^2^ tests were conducted, depending on the curvature of the distribution of the variables' scores.

#### 2.6.2 Qualitative

The accuracy of the transcripts was checked based on the audiotape samples by two of the authors (a social scientist and a clinical psychologist). Data were imported to F4 Analysis^28^ for content analysis.^29^ In F4 Analysis, the audiotapes of the interviews were automatically converted into text. Then, both researchers listened to the tapes and checked the transcripts independently. Relevant clusters (content‐wise) were developed and presented to the steering group of MenDis‐CHD for interpretation. Data and coding within the different clusters were further analysed, named and defined as: (1) perception of cardiac event; (2) patient's needs; (3) access and barriers to the healthcare system and (4) specific age‐related issues. These categories reflect topics most important to patients during the semi‐structured interview. They were identified through discussion until an agreement was reached. Besides creating an overview of relevant content per category, this procedure was chosen to verify that patients would indeed talk about the research questions, as it was the aim to gather more qualitative information on them. Regular in‐depth discussion meetings of the steering group of MenDis‐CHD increased the reliability of the research results.

## 3 RESULTS

### 3.1 Sociodemographic and clinical characteristics

Most participants were male (*n* = 258, 70.9%). The mean age across genders was *M*_age_ = 65.9 (SD = 11.4); 72.5% lived together with a partner (*n* = 264); 27.5% had a left ventricular ejection fraction of <40%; 63.2% had a PCI for a myocardial infarction; 52.5% (*n* = 191) received a positive HADS score and 28.0% (*n* = 102) had a positive SCID diagnosis. Twelve patients had a positive HADS score but refused further testing (SCID‐I). The following SCID diagnoses were found: unipolar depression (*n* = 59, 16.5%), bipolar disorder (*n* = 2, 0.6%), anxiety disorder (*n* = 26, 7.2%), substance use/addiction disorder (*n* = 14, 4.0%), adjustment disorder (*n* = 11, 3.0%) and posttraumatic stress disorder (*n* = 4, 1.1%). Table 1 provides an overview of the demographic and clinical characteristics. For more details on the ICD‐10 diagnoses, see Table A1.

**Table 1:** Sociodemographic and clinical characteristics of the participants

	Patients with MHI (*N* = 102), *n* (%)		Patients without MHI (*N* = 262), *n* (%)		Total (*N* = 364), *n* (%)		*p* Value
Gender							.002
Male	60	58.8	198	75.6	258	70.9	
Female	42	41.2	64	24.4	106	29.1	
Age							.001
35–49 years	9	8.8	15	5.7	24	6.6	
50–59 years	30	29.4	59	22.5	89	24.5	
60–69 years	39	38.2	72	27.5	111	30.5	
70–79 years	10	9.8	80	30.5	90	24.7	
80–95 years	14	13.7	36	13.7	50	13.7	
Marital status							.292
Living together	70	68.6	194	74.1	264	72.5	
Living alone	32	31.4	68	25.9	100	27.5	
Professional qualification^a^							.245
None	14	13.7	19	7.3	32	8.8	
Apprenticeship	56	54.9	149	56.9	176	48.4	
Vocational school	15	14.7	46	17.6	58	15.9	
College/university	16	15.7	53	20.2	66	18.1	
Other	10	9.8	22	8.4	32	8.8	
Retired	48	47.1	143	54.6	191	52.5	.197
Somatic comorbidity^a^							b
Peripheral arterial disease	9	8.8	24	9.2	33	9.1	
Congestive heart failure	25	24.5	77	29.4	102	28.0	
Transient ischaemic attack/stroke	5	4.9	22	8.4	27	7.4	
Cancer	2	2.0	–	–	2	0.5	
Left ventricular ejection fraction							.597
>40%	73	71.6	191	27.1	264	72.5	
≤40%	29	28.4	71	72.9	100	27.5	
NYHA							.001
NYHA I	26	25.5	101	38.5	127	34.9	
NYHA II	41	40.2	115	43.9	156	42.9	
NYHA III	35	34.3	46	17.6	81	22.3	
Myocardial infarction (+PCI intervention)	67	65.7	163	62.2	230	63.2	.960
Bypass surgery	21	20.6	47	17.9	68	18.7	.560
Cardiac valve surgery	6	5.9	18	6.9	24	6.6	.733

Abbreviations: MHI: mental health issue; NYHA; New York Heart Association; PCI, percutaneous coronary intervention.

^a^ It is possible for patients to have more than one answer.

^b^ At least one cell was too small for the appropriate analysis.

### 3.2 Patients' needs

A total of 80.8% with MHI and 74.2% without MHI found it appropriate to have been approached by the doctor (e.g., GP or cardiologist) about MHI; several patients were unsure whether they found it appropriate (5.8% with MHI; 7.5% without MHI). Patients expressed the general wish to be approached proactively by their doctor about MHI (46.5% with MHI; 38.0% without MHI). Approximately a fifth of patients in both groups were unsure about whether they wished to be approached by the doctor about MHI. About 23.4% of MHI patients mentioned a preference for GPs to help them find adequate treatment for drug therapy. For detailed information, see Table 2.

**Table 2:** Details to access, barriers and needs regarding healthcare in CHD patients with and without mental health issues (MHI)

	Patients with MHI (*N* a = 102), *n* (%)		Patients without MHI (*N* a = 262), *n* (%)		Total (*N* a = 364), *n* (%)		*p* Value
Patients' needs							
Wish to be approached by the doctor on MHI	40	46.5	87	38.0	127	34.9	.224
Who should give advice on treatment^b^							.245
General practitioner	57	79.2	38	76.0	95	26.1	
Clinic	6	8.3	7	14.0	13	3.6	
Rehabilitation clinic	12	16.7	9	18.0	21	5.8	
Cardiologist	13	18.1	15	30.0	28	7.7	
Other	9	12.5	6	12.0	15	4.1	
Preferred treatment option							.001
Medication	7	9.6	3	6.0	10	2.7	
Psychotherapy	36	49.3	13	26.0	49	13.5	
Both	15	20.5	9	18.0	24	6.6	
Other	3	4.1	2	4.0	5	1.4	
Wish for treatment for MHI	38	52.8	5	10.4	43	11.8	<.001
Deemed usefulness of psychotherapy in patients							<.001
Not receiving psychotherapy	24	43.6	5	10.6	29	8.0	
Deemed usefulness of medication therapy in							.226
Patients not receiving medication therapy	4	6.8	1	2.1	5	1.4	
Access and barriers to healthcare							
Sufficiently received information on the content and accessibility of *psychological* care							.079
Yes	30	42.3	13	30.2	43	11.8	
Only information about the content of psychotherapy	5	7.0	1	2.3	6	1.6	
Only information on procuring a therapy	6	8.5	1	2.3	7	1.9	
No information	19	26.8	15	34.9	34	9.3	
Information received by							.383
General practitioner	28	50.0	9	30.0	37	10.2	
Clinic	4	7.1	5	16.7	9	2.5	
Rehabilitation clinic	7	12.5	3	10.0	10	2.7	
Cardiologist	–	–	2	6.7	2	0.5	
Health insurance	3	5.4	1	3.3	4	1.1	
Other	15	26.8	4	13.3	19	5.2	
Desired information about psychotherapy provided by							.397
General practitioner	22	47.8	15	44.1	37	10.2	
Clinic	4	8.7	4	11.8	8	2.2	
Rehabilitation clinic	5	10.9	4	11.8	9	2.5	
Cardiologist	4	8.7	4	11.8	8	2.2	
Health insurance	4	8.7	4	11.8	8	2.2	
Other	1	2.2	4	11.8	5	1.4	
Doctors positively respond to patients' wish for psychotherapeutic treatment	33	47.1	6	14.6	39	10.7	<.001
Help with search for psychotherapeutic treatment	27	38.6	6	14.6	33	9.1	<.001^c^
If yes, by whom							
General practitioner	21	47.7	7	22.6	28	7.7	
Clinic	3	6.8	3	9.7	6	1.6	
Rehabilitation clinic	2	4.5	–	–	2	0.5	
Cardiologist	1	2.3	3	9.7	4	1.1	
Family	7	15.9	1	3.2	8	2.2	
Friends	5	11.4	1	3.2	6	1.6	
Colleagues	–	–	–	–	–	–	
Other	6	13.6	–	–	6	1.6	
Waiting period for psychotherapy							.001^c^
1–2 weeks	9	12.9	1	2.4	10	2.7	
3–4 weeks	9	12.9	2	4.9	11	3.0	
2 months	11	15.7	–	–	11	3.0	
3–4 months	6	8.6	–	–	6	1.6	
>4 months	2	2.9	2	4.9	4	1.1	
Sufficiently preserved information on the content and accessibility of *psychiatric* care							.003
Yes	20	28.6	5	12.2	25	6.9	
Only information about content of psychotherapy	4	5.7	1	2.4	5	1.4	
Only information on procuring a therapy	5	7.1	2	4.9	7	1.9	
No information	19	27.1	7	17.1	26	7.1	
Information received by							.013
General practitioner	25	48.1	5	13.9	30	8.2	
Clinic	4	7.7	6	16.7	10	2.7	
Rehabilitation clinic	4	7.7	3	8.3	7	1.9	
Cardiologist	1	1.9	3	8.3	4	1.1	
Health insurance	3	5.8	1	2.8	4	1.1	
Other	12	23.1	3	8.3	15	4.1	
Desired information about drug therapy provided by							.201
General practitioner	25	49.0	9	23.7	34	9.3	
Clinic	7	13.7	5	13.2	12	3.3	
Rehabilitation clinic	7	13.7	3	7.9	10	2.7	
Cardiologist	3	5.9	3	7.9	6	1.6	
Health insurance	7	13.7	3	7.9	10	2.7	
Other	2	3.9	1	2.6	3	0.8	
Doctors positively respond to patients' wish for drug therapy	17	24.3	1	2.4	18	4.9	002
Help with search for drug therapy	13	18.6	2	4.8	15	4.1	.070^c^
If yes, by whom							
General practitioner	11	23.4	2	7.1	13	3.6	
Clinic	2	4.3	2	7.1	4	1.1	
Rehabilitation clinic	–	–	1	3.6	1	0.3	
Cardiologist	2	4.3	1	3.6	3	0.8	
Family	3	6.4	–	–	3	0.8	
Friends	2	4.3	–	–	2	0.5	
Colleagues	–	–	–	–	–	–	
Other	2	4.3	–	–	2	0.5	
Waiting period for an appointment with the psychiatrist and/or psychotherapist							c
1–2 weeks	5	7.1	–	–	5	1.4	
3–4 weeks	6	8.6	1	2.3	7	1.9	
2 months	2	2.9	–	–	2	0.5	
3–4 months	4	5.7	–	–	4	1.1	
>4 months	1	1.4	–	–	1	0.3	

Abbreviation: CHD, coronary heart disease.

^a^ Multiple‐choice question.

^b^ All percentages relate to the maximum number of patients who were eligible for the underlying question.

^c^ At least one cell was too small for the appropriate analysis.

### 3.3 Access and barriers to healthcare

A total of 30 CHD patients with MHI (42.3%) stated that they received an adequate amount of information on the content and accessibility of psychological care. Only a minority of patients obtained information about psychotherapy (7.0%) with a quarter receiving no information at all. About 30% of patients obtained information on the content and accessibility of psychological care through their doctors, while 35% stated that they did not receive further information. When patients did obtain information, half of all patients with MHI got information from the GP or other persons outside of the healthcare system (e.g., family members, friends); 47.8% of MHI patients and 44.1% of patients without MHI wished to get this specific information from the GP. For further information, see Table 2.

### 3.4 Qualitative

In total, 65 CHD patients agreed to participate in a qualitative interview. These patients were mostly men (*n* = 53) and had an ejection fraction of <40% (*n* = 41). Divided over the three settings, 21.5% of patients came from hospitals, 33.9% from rehabilitation clinics and 44.6% from cardiology practices. From this subpopulation, 20 patients were selected and asked to participate. The mean age was *M* = 67.2 (SD = 12.6). All patients lived with a partner. For detailed information about the selection process, see Figure 1.

### 3.5 Perception of the cardiac event and approaches by doctors to talk about MHI

The following section contains information about how the patients experienced the cardiac event and whether they were really approached by the doctor to talk about MHI. Patients experienced CHD as a decisive turning point that unsettled them permanently. Most respondents felt significant anxiety and uncertainty in the weeks following the cardiac event. After diagnosis, spouses and family were the central persons to whom patients articulated their fears. Only a few patients reported that they were approached about MHI by a doctor as part of the CHD diagnosis. In general, MHI was not discussed until months after the diagnosis. Few patients engaged in a conversation about MHI with a professional. They reported that either the GP or a psychologist in a rehabilitation clinic approached them.

No, not really. No one actually asked, no doctor, whether there was still a need for treatment. Patient 16, l. 22–24

Of course, the doctor should have to ask more background information. How the patient is doing in other areas after this disease, whether he has psychological issues. I think that most specialists, e.g., cardiologists, proceed very technocratically and reel off their plan and don't care much about other things. I have not met any cardiologist, except for the head physician at the university hospital, who has dealt more intensively with this issue. For them it is only important how the echography is, how the ultrasound examination is, the blood values, etc. That's it. I even believe that cardiologists don't have a heart (laughs). Patient 9, l. 78–85

Well, I have the feeling that my cardiologist doesn't get involved with me on an emotional level. It's relatively quick, he does a ECG or an ultrasound or something else and then I get my information and have to leave. It's not something where you have a longer conversation, that's what they do. only the treatment, then you go back to the treating GP. Patient 14, l. 131–137

### 3.6 Patients' needs

#### 3.6.1 The wish for a conversation about MHI

All patients reported that they would find it appropriate to be asked by a doctor about MHI and that they wish for a conversation about MHI with their doctors. Patients reported a high level of suffering until they talked about MHI. If patients had a professional talk about MHI, the important counterpart was the GP. The conversation was experienced as very positive and relieving when GPs talked about psychological problems on their initiative. Patients reported that the professional talk about MHI should occur shortly after the CHD diagnosis. Patients deliberately changed the GP to find a doctor who was willing to talk about MHI or even actively approached them about possible MHI. One patient experienced contact with his cardiologist as very technical and rational and switched to a cardiologist where he also experienced emotional care. Few patients remembered that they were approached for MHI during their stay in rehabilitation clinics. Those who had psychological appointments in rehabilitation clinics experienced them as positive. No further psychological treatment was needed in the subsequent treatment. Interventions had positive effects on patients' coping skills and self‐care but also resulted in feelings of relief.

I think that all this belongs to a good doctor. Of course, he should carefully address everything. But he should ask. Everything, all these things, so that a depression can be recognized at an early stage. […] In the course of the conversation the doctor might get the impression that there is a serious problem and I think a good doctor should also address that. Patient 8, l. 68–70

It has to be a doctor, a GP, who talks to people. So, we've had a new family doctor for almost two years now, and he talks to me. The previous one didn't talk to me. And I'm very happy that I can go there, where I can just sit for a moment and think about what I still have to talk about, and as far as I know that's not usual. So otherwise, I came in, five minutes,—bang bang—went out of the room, got my prescription and that's it. I just don't want to have things like that anymore. Patient 14, l. 215–222

But then there was a doctor in rehab, that was the first one who really listened to me, and he also got my story, really informed himself, he was the one who told me that everything was connected. The depression, the heart, and so on. This man has helped me very much. He acknowledged that. that this was not overreacting. I was really suffering. Patient 8, l. 206–210

#### 3.6.2 No wish for a conversation about MHI

A minority of patients did not want to talk about possible psychological distress and denied it. They reported strictly separate physical and psychological impairments or even denied an interaction between the two. One patient denied a link between physiological events such as CHD and possible psychological consequences.

No. For me, everything just continues as before. The heart attack was basically like a car repair. There was a doctor who said I had a constriction in the arteries of the heart where this stent was inserted. And after that, that was it for me, I hardly gave it a second thought. Patient 5, l. 68–72

#### 3.6.3 Access and barriers

Limited resources (e.g., lack of time, staff shortage) were often reported as barriers. Several interviewed patients reported that during a triweekly stay in a rehabilitation clinic, only a few patients were screened and asked about MHI. Patients with MHI reported numerous unsuccessful attempts to have a psychological appointment and failures to find an ambulant psychotherapist. They complained about insufficient support by the healthcare system, lacking opportunities to talk about their MHI on medical appointments and lacking help with the search for an ambulant psychotherapist. Drug therapy was not a sufficient alternative for this group; most of them rejected this alternative. Patients were demoralized by those failures, hence they developed a passive coping style, stopped actively searching for psychotherapeutic support or postponed it for the future.

The rehabilitation was initiated in the hospital and in the rehab itself there also was a psychologist who was supposed to take care of the processing of these things, but unfortunately she was ill while I was in rehab. And then I was not able to implement this in the rehab. I would have liked to have some appointments with her. Now I had bad luck that the psychological support was cancelled. Maybe that would have changed something. Patient 9, 22–26, l. 197–200

Well, a drug therapy. I don't believe that this is necessary. Maybe in one case for sure, but I don't think that would help me. Patient 16, l. 94–96

#### 3.6.4 Specific age‐related issues

Besides the three main categories, a fourth important category emerged and was summed up as ‘specific issues of younger and older patient groups’.

#### 3.6.5 The younger patient group and their concerns about their ability to work

The needs of patients varied according to their life phases. Patients in the age range 50–60 years expressed fears and concerns about their future ability to continue with their work. They were also the subgroup who experienced the most depression and anxiety symptoms and reported the highest level of suffering. Employed patients were more accessible to talk about MHI in general, were more sensitive to handle the situation adequately, showed a high adherence to medical advice, were more informed about possible psychotherapeutic services, were more motivated to be active in their medical treatment and had better problem‐solving mechanisms.

Well, I'm working again now, aren't I? But when it came to the question of when I would go back to work, I was very unhappy and very uncertain and I thought, how can I do that? Because it is … well, it is still that I am anxious. So, I have body sensations that are strange to me. And they frighten me. And then I think, well, I hope I don't fall down and die. Well, that's something like that. And once I wasn't feeling well and my GP said to me: ‘You can live up to a 100 years with the bypass, but if you don't work on yourself or something like that, then it's not good for your heart and you'll get sicker.’ […] I'm concerned about the disabled person's pass. Maybe I should go to a psychiatrist to discuss it. Patient 14, l. 37–51

I don't know if you can imagine that you would be told overnight that you can no longer participate in the active working life that you had and could have until then. Patient 7, l. 48–50

#### 3.6.6 The older patient group and their concerns about successful aging

On the other side, patients who were already retired (65 years and above) expressed more worries about successful ageing. This type of patient often reported fears about the future quality of life and the responsibility for their own family. Retired patients suffered from more somatic comorbidities and were less active in their own medical treatment. For such patients, social networks functioned as a resistance factor. Other factors were being optimistic, acceptance of the current situation and appreciating the last years of life.

‘I think the family must be informed, especially the closer family circle. Just because I had two heart attacks and I hear badly on the left side. that is a handicap in life that you don't see. […] these are limitations that no one can imagine […] this handling of heart diseases in society … if you are no longer so efficient and so resilient, mentally as well as physically, it really is an impairment. One does not see the disease and it is no problem for an outsider who has no problems with it … (Those people) think to themselves: why can't this man walk five stairs? […] And these are such things that are important for a family, because one must also manage everyday life and have the support to get along again in social life. Patient 16, l. 117–125 and l. 135–144

## 4 DISCUSSION

This mixed‐method study aimed at exploring needs, access and barriers in the care of CHD patients from the patient's viewpoint. In our study, 28% of CHD patients were identified as having MHI. In accordance with the literature, the MHI in our study were mainly depression and anxiety disorders,^7^, ^8^, ^30^ and women were twice as likely to develop MHI.^1^ Taking the quantitative and qualitative results together, patients reported unmet needs, decreased access to helpful resources and encountered multiple obstacles in handling their MHI adequately.

### 4.1 Perception of the cardiac event and patients' needs

Both quantitative and qualitative data show in general that for all recruitment centres (hospital cardiac departments, cardiology practices and rehabilitation clinics) patients with and without MHI experience anxiety and uncertainty after a cardiac event. This event was perceived as drastic and life‐changing, and consequently, patients felt a great need to talk about what had happened. They expressed disappointment about the fact that doctors rarely address MHI. Interviewed patients stated that they show little initiative during their medical consultation and usually did not address MHI on their own. A study from Lussier et al.^11^ developed an online tool to encourage patients to be more active and prepared for their healthcare encounters by providing skills coaching via a website. Although patients reported a positive perception of such coaching and a higher motivational level, barriers such as lack of interest, limited access to technology, lack of time or language skills reduced the success of this approach.^11^

However, when MHI was discussed with the doctor, patients did report a decrease in symptoms, mainly a reduction in anxiety, and a positive influence on their feeling of being able to cope with the cardiac event. It therefore seems reasonable that doctors, for their part, ask more regularly about MHI during medical consultations to improve the previous deficit in the area of emotional care. For this purpose, it seems necessary to have targeted interventions, such as creating greater awareness for this topic and the vulnerable patient group in everyday medical practice, to ensure rapid detection of MHI and thus adequate care. Further training courses, in the field of basic psychosomatic care and medical interviewing, could be helpful to increase individual communication skills in doctor–patient conversations, especially in an empathic manner during medical conversations. Studies showed that empathy of the doctor perceived by the patient significantly improved patient satisfaction with the received medical care, especially in terms of information exchange, treatment, perceived expertize of the doctor, as well as interpersonal trust and partnership with the doctor.^31^ A higher empathy‐related behaviour of the doctor might be useful to improve the doctor–patient relationship and might increase the chance of patients daring to speak about MHI to their doctor.

### 4.2 Access and barriers to healthcare and specific issues of patient groups

In general, patients received little support in seeking psychological, psychiatric or psychotherapeutic help. Lack of time and personal opportunities, none to little help in coping with mental stress, and finding an ambulant psychotherapist were found to be barriers for patients and GPs in dealing with their CHD‐related MHI's.

Shortcomings in the early stages of CHD and MHI‐related treatment can lead to demoralization and learned helplessness in patients. At the same time, complaints such as future‐ and health‐related worries, anxiety, depression and fear of incapacity for work continue to exist and further restrict patients' mental and physical health. As mentioned before, MHI and even mental stress are associated with higher CHD‐related morbidity and mortality^7^ and hinder the required lifestyle changes to reduce CHD risk factors.^4^, ^8^

These shortcomings in medical healthcare can occur because the current healthcare system defines ‘valuable care’ as health outcomes relative to costs and hence encompasses efficiency. However, cost reduction without regard to the achieved outcomes leads to limited effective healthcare.^32^ Following Porter,^33^ *value‐based healthcare* has the following definition: *value* equals *health outcomes* that matter to patients regarding the *costs* of delivering the outcomes. Thus, value measures all services and activities that determine success by meeting the patients' needs. These needs are determined by the medical condition of the patient and are an interrelated set of medical circumstances. A medical condition includes the most commonly associated conditions—meaning that, for example, care for CHD patients must consist of care for hypertension, and so forth.^32^, ^33^ Regarding the high prevalence of MHI in CHD patients, it might be advisable to include MHI as further interrelated medical circumstances. For example, only CHD patients in rehabilitation clinics reported having access to psychotherapeutic help in general; patients from practices and hospitals lacked this possibility. Rehabilitation clinics seem to work more within a more value‐based healthcare cycle because they are medical units that specialize in several diseases and thus are organized in the same way as an integrated practice unit,^33^ thus including all the necessary skilled medical practitioners needed for medical conditions and their co‐occurring problems.

To deal with the shortcomings found in this study, a more value‐based and patient‐centred approach is necessary. With regard to structural requirements, earlier detection and correct diagnosis of MHI, as well as appropriate and fast treatment, would be examples (i.e., an appointment with an outpatient psychotherapist). In addition, more psychological measures, such as training for doctors (e.g., to increase the level of empathy and better support in coping with CHD and MHI) and improvements in procedural requirements (more conversation time during doctor contacts and more precise agreements between different departments) are needed. To be able to implement such requirements in practice adequately, the deployment of nursing or case managers in GP practices would be advisable. Nursing managers could use low‐threshold interventions to assist CHD patients with MHI in the search for a suitable form of psychotherapy and/or drug therapy and to accompany their implementation. They could act as a link between the various care providers and ensure a timely exchange of information and early personal appointments.^34^

### 4.3 Strengths, limitations and further research

MenDis‐CHD provided insights into the needs of CHD patients with and without MHI and their access and barriers to healthcare, clarified important health outcomes for this population and indicated the requirements that a more value‐based care system should focus on to increase quality and efficiency.

Nonetheless, this study has some limitations. With regard to the qualitative part of the study, we chose to conduct 20 interviews. Looking at the pool of eligible patients, our sample size is limited by the participant diversity and the recruitment location (e.g., recruitment only in one city in Germany and particularly in cardiology practices). Because patients were mainly recruited in outpatient settings, it is possible that they were biased with regard to their preference to talk to a GP about their MHI. Patients may have perceived the outpatient setting as a familiar environment, as there has not necessarily been an inpatient stay recently and could therefore adopt a one‐sided view. Another limitation was that some of the quantitative items in our questionnaire were self‐developed and had no statistical testing of validation. Replication with a larger sample size and greater diversity, either across Germany or internationally, would be advantageous to explore possible generalization effects. Other vulnerable population groups that also have a high prevalence of MHI (e.g., schizophrenia) should be examined to explore specific health outcomes and needs that are important to the particular group of patients. With more data, researchers could build a better basis in clinical practice to facilitate a change towards a value‐based healthcare system.

## 5 CONCLUSION

This study found that the needs regarding CHD patients with and without MHI were not adequately satisfied. Furthermore, for CHD patients with MHI, access and barriers to the healthcare system regarding psychotherapeutic and drug therapy were insufficient. CHD patients experienced the cardiac event as life‐changing and had an urgent need to talk about mental problems. Primarily, the GP was named as a partner for conversations about CHD‐related mental problems; however, when the doctor spoke to the patient shortly after the cardiac event, patients felt relieved and coped better with their illness. To improve this, psychological measures such as training of doctors in doctor–patient communication are necessary, as well as improvements in procedural and structural requirements.

## AUTHOR CONTRIBUTIONS

Samia Peltzer wrote the abstract, introduction, method section, results (incl. analyses) and discussion, designed the figure and tables, references with EndNote, made an overall adaption of the text and included references. Samia Peltzer was a major contributor in writing the manuscript. Ursula Köstler conducted the information for the qualitative analysis, conducted qualitative analysis and summed up the results for the qualitative part. Frank Jessen, Frank Schulz‐Nieswandt and Christian Albus designed and conducted the study as chief scientists. Hendrik Müller, Nadine Scholten, Frank Jessen and Christian Albus critically revised the manuscript. All gave final approval and agreed to be accountable for all aspects of work, ensuring integrity and accuracy.

## Data Availability

The data underlying the results presented in the study are available. The CoRe‐Net database trust center within the Cologne Research and Development Network (CoRe‐Net) is responsible for reading and saving the data. This is located at the Institute for Medical Sociology, Health Services Research and Rehabilitation Science (IMVR) of the Faculty of Human Sciences and the Medical Faculty of the University of Cologne, Eupener Str. 129, 50933 Cologne, Director: Prof. Dr. Holger Pfaff. Prof. Dr. Holger Pfaff is mainly responsible for data processing.

## 1

See Table A1

**Table A1:** Overview of all SCID‐I diagnoses

	Center			Total (*N* = 364), *n*
	Hospitals (*N* = 107), *n*	Rehabilitation clinics (*N* = 157), *n*	Practices (*N* = 100), *n*	
SCID‐I diagnoses^a^				
No SCID diagnosis	82	103	77	262
Refused SCID testing	5	5	2	12
Positive SCID diagnoses	26	61	29	102
Positive SCID‐I diagnoses^a^				
Alcohol dependence in remission (F10.21)	3	3	3	9
Cannabis abuse (F12.1)	–	2	–	2
Cocaine dependence, in remission (F14.21)	–	1	–	1
Multiple substance use disorder (F19.2)	–	–	1	1
Multiple substance‐induced psychotic disorder (F19.5)	–	1	–	1
Bipolar disorder, current episode depressed, moderate (F31.32)	–	1	–	1
Bipolar disorder, most recent episode depressed, in partial remission (F31.75)	1	–	–	1
Major depressive disorder, single episode, mild (F32.0)	1	4	–	5
Major depressive disorder, single episode, moderate (F32.1)	5	7	5	17
Major depressive disorder, single episode, in partial remission (F32.4)	2	5	3	10
Major depressive disorder, single episode, unspecified (F32.9)	1	1	–	2
Major depressive disorder, recurrent, mild (F33.0)	–	3	–	3
Major depressive disorder, recurrent, moderate (F33.1)	2	6	1	9
Major depressive disorder, recurrent, in full remission (F33.42)	1	–	–	1
Dysthymic disorder (F34.1)	–	–	2	2
Depressive disorder (self‐declaration questionnaire)	3	3	4	10
Agoraphobia with panic disorder (F40.01)	–	2	1	3
Agoraphobia without panic disorder (F40.02)	–	–	1	1
Specific phobia (F40.2)	–	2	1	3
Panic disorder (F41.0)	1	–	–	1
Generalized anxiety disorder (F41.1)	1	–	–	1
Anxiety disorder, unspecified (F41.9)	1	1	3	5
Anxiety disorder (self‐declaration questionnaire)	3	6	3	12
Posttraumatic stress disorder (F43.1)	–	4	–	4
Adjustment disorder (F43.2)	1	9	1	11

Abbreviation: SCID‐I, Structured Clinical Interview for DSM‐IV Axis I Disorders.

^a^It is possible for patients to have more than one SCID diagnosis.
